# A proteomic approach to analyzing responses of *Arabidopsis thaliana *root cells to different gravitational conditions using an agravitropic mutant, *pin2 *and its wild type

**DOI:** 10.1186/1477-5956-9-72

**Published:** 2011-11-16

**Authors:** Chao Tan, Hui Wang, Yue Zhang, Bin Qi, Guoxin Xu, Huiqiong Zheng

**Affiliations:** 1 Institute of Plant Physiology and Ecology, Shanghai Institutes for Biological Sciences, Chinese Academy of Sciences, 300 Fenglin Road, Shanghai 200032, China; 2 Institut für Biologie II Botanik, Albert-Ludwigs-Universität Freiburg, Schänzlestress 1, Freiburg 79104, Germany

**Keywords:** proteomics, Annexin, clinorotation, hypergravity, *Arabidopsis thaliana*, *pin2 mutant*, root tip

## Abstract

**Background:**

Root gravitropsim has been proposed to require the coordinated, redistribution of the plant signaling molecule auxin within the root meristem, but the underlying molecular mechanisms are still unknown. PIN proteins are membrane transporters that mediate the efflux of auxin from cells. The PIN2 is important for the basipetal transport of auxin in roots and plays a critical role in the transmission of gravity signals perceived in the root cap to the root elongation zone. The loss of function *pin2 *mutant exhibits a gravity-insensitive root growth phenotype. By comparing the proteomes of wild type and the *pin2 *mutant root tips under different gravitational conditions, we hope to identify proteins involved in the gravity-related signal transduction.

**Results:**

To identify novel proteins involved in the gravity signal transduction pathway we have carried out a comparative proteomic analysis of Arabidopsis *pin2 *mutant and wild type (WT) roots subjected to different gravitational conditions. These conditions included horizontal (H) and vertical (V) clinorotation, hypergravity (G) and the stationary control (S). Analysis of silver-stained two-dimensional SDS-PAGE gels revealed 28 protein spots that showed significant expression changes in altered gravity (H or G) compared to control roots (V and S). Whereas the majority of these proteins exhibited similar expression patterns in WT and *pin2 *roots, a significant number displayed different patterns of response between WT and *pin2 *roots. The latter group included 11 protein spots in the H samples and two protein spots in the G samples that exhibited an altered expression exclusively in WT but not in *pin2 *roots. One of these proteins was identified as annexin2, which was induced in the root cap columella cells under altered gravitational conditions.

**Conclusions:**

The most interesting observation in this study is that distinctly different patterns of protein expression were found in WT and *pin*2 mutant roots subjected to altered gravity conditions. The data also demonstrate that PIN2 mutation not only affects the basipetal transport of auxin to the elongation zone, but also results in an altered expression of proteins in the root columella.

## Background

Gravity plays an important role in the regulation of plant growth and development [1-4]. Shoots and roots of plants orient themselves with respect to the gravity vector, with roots growing towards the gravity vector and shoots in the opposite direction. Underlying this response is a series of complex biological processes that include gravity sensing, signal transduction, signal transmission and the growth response [5-9]. In roots, the primary site for gravity sensing is located in the columella cells of the root cap, the differential growth response occurs in the elongation zone, which, in Arabidopsis, is located at a distance of > 1 mm from the root cap. The classic Cholodny-Went theory proposes that auxin acts as the signal that carries the gravitropic information from the root cap to the elongation zones (reviewed in [10-12]). Transport of auxin across the plasma membrane is mediated by two types of influx carriers (AUXIN1/like Aux family) and efflux (PIN-FORMED family) carriers [13-20]. In gravistimulated organs auxin transport appears to be direct primarily by PIN-type carriers [21-23]. Of the five PIN proteins identified in Arabidopsis, PIN2 and PIN3 have been demonstrated to be directly involved in gravitropsim [11,24,25]. The polar subcellular localization of these PIN proteins in the plasma membrane determines the direction of intercellular auxin flow and thereby the gravitropic growth response [26].

PIN2, which is expressed in the cortical and epidermal cells of the meristematic and elongation zones of roots, is particularly important because it appears to be responsible for the basipetal transport (*i.e*. from the root tip to elongation zone) of auxin in roots [14,16,27]. Any disruption of this basipetal transport of auxin affects the gravitropic response of the roots [28,29]. The loss of PIN2 function in *pin2 *mutant impairs basipetal auxin transport in the roots thereby preventing the elongation zone from responding to the gravitropic stimulus [15,16]. The role of PIN2 in root gravitropism has been studied extensively in Arabidopsis [11,18,30], including the mechanisms that regulate *PIN2 *transcription, and the subcellular localization and degradation of PIN proteins in gravity sensing root tips [31]. These previous studies have provided us a comprehensive view on the transportation of auxin from the site of gravity perception to the growth response region. Still lacking is information on which proteins besides the auxin efflux facilitator PIN2 are involved in this process.

In inflorescence axes the polar transport of auxin is decreased when Arabidopsis plants are grown on a horizontal clinostat [32]. Although less is known about the changes in the polar transport of auxin in roots in response to altered gravity conditions, microarray and proteomic analysis in Arabidopsis root tips and callus cultures have demonstrated that rapid reorientation of seedlings alter the expression of both genes and proteins [33-39]. In a previous study, we have studied changes in the proteome of Arabidopsis callus cells in response to clinostat rotation that randomized the orientation of the gravity vector [40]. The data presented in that study showed that clinostat rotation of Arabidopsis callus cells had a significant impact on the expression of proteins involved in general stress responses, metabolic pathways, gene activation/transcription, protein synthesis, and cell wall biosynthesis.

Here we report on a comparative proteomic analysis of responses of Arabidopsis wild type and *pin2 *mutant roots to different gravitational conditions, including horizontal and vertical clinorotation and hypergravity, all in comparison to a stationary control. The functional implications of the observed changes in protein expression in response to clinorotation and hypergravity are discussed. Furthermore, the determination of the cell-type specific expression of annexin2::GFP, a calcium-related protein, was performed.

## Results and Discussion

**Figure 1:**
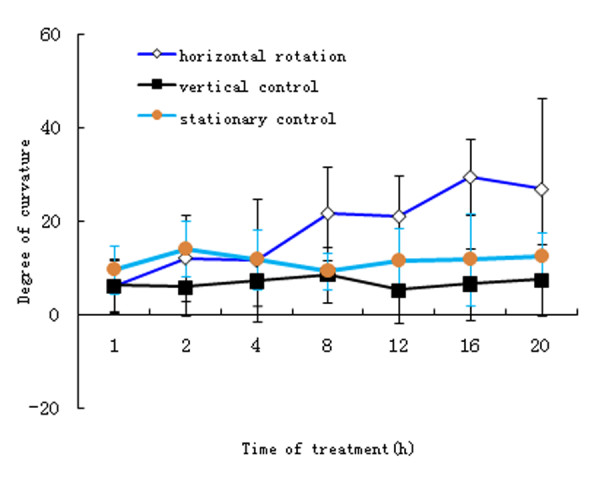
**Analysis of curvature responses of roots of six-day old Arabidopsis wild-type seedlings exposed for 1, 2, 4, 8, 12, 16 and 20 h to a horizontal clinorotation, a vertical clinorotation, in relation to a 1 g stationary control, respectively**. Data are mean±SD, n > 50 roots.

To study the directional and magnitude effects of gravity on plants and identify the gravity-regulated proteins that function in the gravity response mechanism (Additional file 1 Figure S1), we have investigated six-day old Arabidopsis wild-type (WT) and *pin2 *mutant seedlings subjected to one of the following four treatments: (1) clinorotation at 5 rpm for 12 h along their horizontal axis in a horizontal clinostat (H, simulated weightlessness); (2) clinorotation at 5 rpm for 12 h along the vertical axis in a vertical clinostat (V, clinostat control); (3) centrifugation for 30 min in a low-speed centrifuge to impose hypergravity (7 g)(G); (4) a stationary, control environment(S, ground gravitational force). As reported previously, *pin2 *roots exhibited a defective gravitropic response when grown under S conditions compared to WT roots (Additional file 1 Figure S2, a and b) [14-16]. The curvature responses of Arabidopsis WT roots under the H, V, or S condition as well as re-orientation by 90° under 1 g (S) condition were measured. The positive gravitropic curvature of WT roots reached ~ 70° at 12 h and increased to ~90° at about 16 h after re-orientation under 1 g condition (Additional file 1 Figure S3). The differential growth responses of WT roots subjected to the horizontal clino-rotation were reduced to about 20° at 5 rpm for 12 h (Figure 1). Although the horizontal clino-rotation caused a slight increase in the mean curvature of the roots, there was no statistically significant difference from those under the V or S condition (Figure 1). These results indicate that a positive gravitropic response of WT roots was suppressed by the horizontal clino-rotation. In addition, no phenotypic differences were observed among WT plants grown on the H, V (Additional file 1 Figure S2, d and f) or G condition (data not shown) in comparison with the S control (Additional file 1 Figure S2, b). *pin2 *roots, which showed no positive gravitropic response under the S control condition (Additional file 1 Figure S2 a), exhibited automorphosis-like growth under the H, V (Additional file 1 Figure S2 c and e) or G conditions (data not shown).

**Figure 2:**
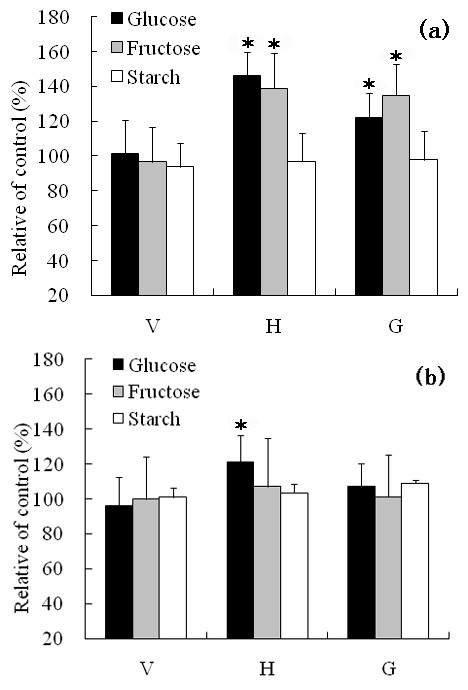
**Differences in sugar and starch concentration in Arabidopsis WT (a) and the agravitropic mutant *pin2 *(b) root tips subjected to either horizontal or vertical clinorotation or a hypergravity treatment compared with the control roots grown under stationary condition**. All clinostat rotational treatments were at 5 rpm for a period of 12 h, and hypergravity treatments were at 7 g for 30 min. H, horizontal clinorotation; V, vertical clinorotation; G, hypergravity. Data represent the mean of 5 replicates, each replicate consisting of a separate extraction of 20 root tips. Statistically significant differences at the 5% level of significance are indicated by asterisk. Bars designate standard deviation.

Previous studies have shown that microgravity conditions in space, randomization of the gravity vector by clinorotation, and creation of hypergravity conditions by centrifugation all affected the sugar concentration and the metabolic fluxes of plant cells [35,41,42]. To further characterize the effects of altered gravity conditions on root cells of WT and *pin2 *plants, we have also compared the changes in concentration of glucose, fructose and starch in WT and *pin2 *mutant roots (Figure 2). The concentration of glucose in WT roots increased by 46% and 22% respectively after a 12 h period of H-treatment and a 30 min period of G-treatment, while fructose increased by 39% and 35%, respectively, under the same conditions. However, the amount of starch remained statistically unchanged in the H or G compared with the S or V-treated samples (Figure 2a). No significant differences were observed in the sugar and starch content of the *pin2 *mutant roots subjected to H, V, G, and S treatment conditions, except for a slight increase in glucose in the H-type roots (Figure 2b). These results indicate that the hexose metabolism of *pin2 *roots is insensitive to altered gravity in comparison to WT. To gain further insight into the molecular basis of these different responses to altered gravity, we have compared the proteomes of Arabidopsis WT and *pin2 *mutant root tips subjected to horizontal clinorotation or hypergravity with those of V and S-treated roots.

### Quantitative proteome differences between pin2 and WT roots under altered gravitational conditions

**Figure 3:**
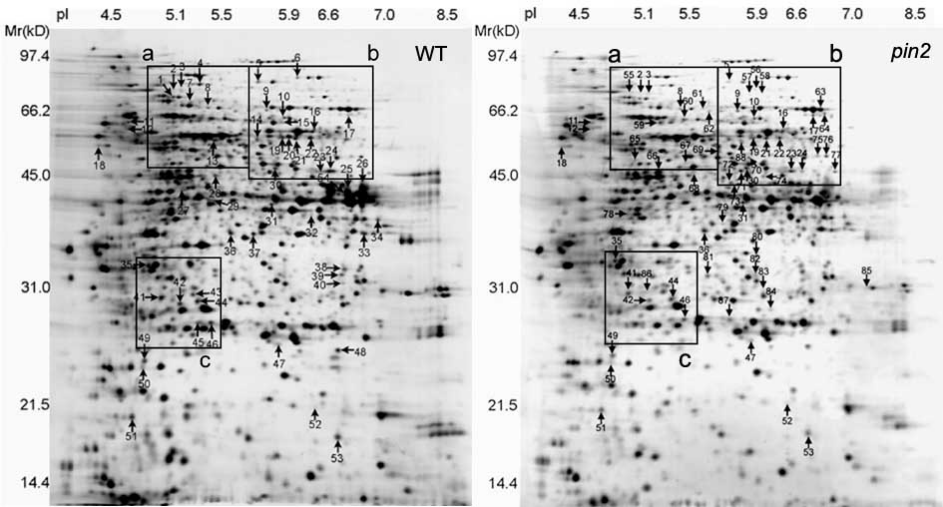
**Images of 2-D gels of proteins extracted from Arabidopsis wild type (WT) and *pin2 *mutant (pin2) roots tips exposed to either clinostat rotation or hypergravity**. The proteins were visualized by silver staining. The arrows indicate 54 protein spots in the WT (left panel) and 64 protein spots in the *pin2 *mutant (right panel) root tips that changed in abundance and/or position after at least one treatment of vertical clinorotation, horizontal clinorotation, and hyper gravity centrifugation. The sum number of the protein spots in both gels is 88 including 30 overlaps. The background gel was of a stationary control. The ranges of pI and molecular masses (kDa) are indicated. The framed regions a, b, and c are detailed in Figure 4.

**Figure 4:**
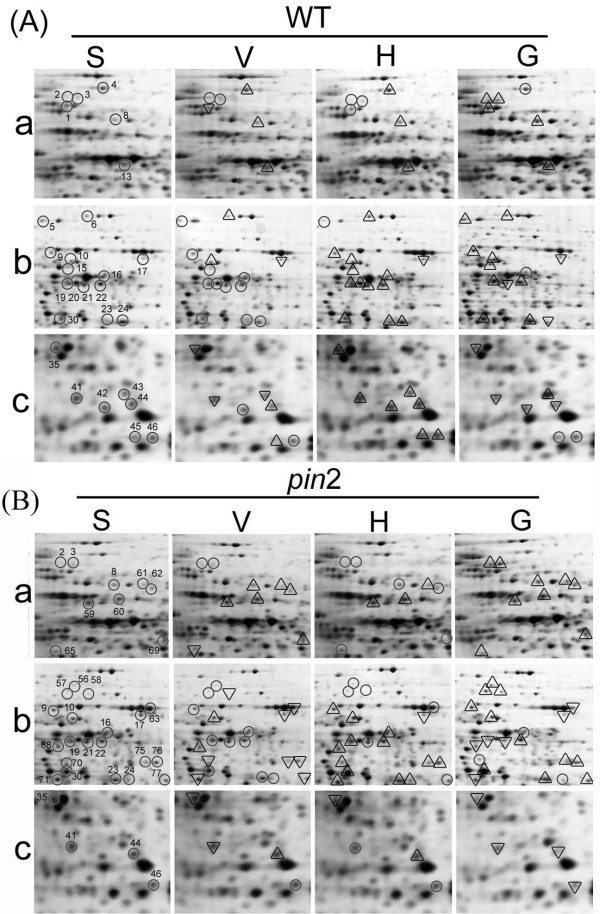
**Enlargement of selected regions in Figure 3 to highlight some of the differentially expressed protein spots whose abundance changed in Arabidopsis in wild type (WT) (A) and *pin2 *mutant (*pin2*) roots tips (B) after exposure to either vertical clinorotation (V) or horizontal clinorotation (H) or hypergravity (G) compared with those that remained stationary control (S)**. a, b, and c correspond to the framed regions a, b, and c in figure 3, respectively. Spot numbers correspond to those shown in the reference gels. Circles, triangles and reversed triangles indicate the spots with no change, increased and decreased intensity, respectively, in the V, H and G samples compared to the S samples.

Root tip regions, 5-10 mm long, were excised immediately after being subjected to H, V, G, or S-treatments. Total proteins in the root tips were extracted and analyzed by two-dimensional (2-D) gel electrophoresis with IPG gels in the first dimension and the resulting images were analyzed using PDQuest 2-D software. The proteomic profiles of WT and *pin2 *root tips grown under S-control conditions are very similar as shown in Figure 3. About 1300-1500 protein spots were reproducibly detected and reliably quantified in silver-stained 2-D gels. Comparisons were preformed among H, V, G and S treated samples, respectively (Figure 3). Selected parts of the gels are highlighted in Figure 4 to illustrate some of the changes in specific protein spots. Statistical analysis showed that in WT roots 54 protein spots were altered either in their abundance and/or pI (P < 0.05) in H, V, or G gels compared to S gels(Additional file 1 Table S1). In the *pin2 *roots the number of altered protein spots was 64 (Additional file 1 Table S2), and the total number of altered proteins identified in these experiments was 88 including 30 overlaps (Figure 3). Of these 88 differentially responding proteins, twenty-eight were increased or decreased in abundance and/or changed in position in the H or the G samples. In contrast, no statistically relevant differences in protein spots between the V and S samples were detected (Additional file 1 Table S1 and S2), indicating that simply rotating the seedlings in the vertical orientation had little effect on the expression of these proteins. These 28 protein spots were analyzed by micro high performance liquid chromatography-ion trap-mass spectrometry (LC-IT-MS) to obtain the tandem mass (MS/MS) spectra. Twenty-five different proteins were identified with high confidence using SEQUEST with uninterpreted MS/MS raw data. The identified spots with their protein coverage, etc. in the clinorotated (H) or hyper g-force (G) treated samples of both WT and *pin2 *mutant are presented in Table 1 and Table 2 respectively. According to the differential expression patterns in WT and/or in *pin2 *roots in response to the H and/or the G treatment, these proteins can be divided into three groups.

**Table 1:** Identified proteins whose expression levels changed between *Arabidopsis thaliana *wild-type and *pin*2 root tips subjected to horizontal clinorotation compared to those under stationary control using two-dimensional electrophoresis and liquid chromatography- ion trap mass spectrometry

Spot No^a)^	Obs.^b)^ Mr/pI	Identity of the proteins	**No**. Mat^d)^	% Sq Cov^e)^	Theo.^c)^ Mr/pI	**Acces**. no.^f)^	H/S^g)^	
							WT	*pin2*
		**Group I**						
9	65/5.66	TCP-1 chaperonin-like protein	14/10	23.52	59/5.97	CAC01806.1	1.5	2.2
22	54/6.00	ATP synthase beta chain 2, mitochondrial	47/21	53.42	60/6.18	sp[P83484]	1.7	1.7
23	44/6.12	Mevalonate diphosphate decarboxylase	3/2	8.98	46/6.33	CAA76803.1	1.8	2.1
30	45/5.72	Isocitrate dehydrogenase	60/20	50.24	46/6.13	CAD24782.1	2.3	2.4
19	54/5.76	Aldehyde dehydrogenase (NAD^+^)-like protein	4/4	12.08	58/7.11	CAB41139.1	2	2.5
24	44/6.42	Glutathione-dependent formaldehyde dehydrogenase class III ADH	24/9	31.93	41/6.51	CAA57973.1	1.9	2.5
16	57/6.08	Putative inosine-5'-monophosphate dehydrogenase	9/6	21.07	54/6.34	sp[Q9SA34]	2.6	2.1
47	25/5.73	Glutathione S-transferase 6	3/2	17.67	24/6.09	sp[Q96266]	1.9	3.3
		**GroupII**						
15	60/5.77	Putative malate oxidoreductase	21/13	33.00	66/6.85	CAB80866.1	2.4	NC
21	54/6.00	ATP synthase alpha chain, mitochondrial	89/20	42.60	55/6.23	sp[P92549]	2.0	NC
20	55/5.79	Enolase(2-phosphoglycerate dehydratase)	24/13	52.03	48/5.54	sp[P25696]	1.8	NC
25	41/6.66	Glyceraldehyde-3-phosphate dehydrogenase	44/14	62.13	37/6.62	sp[P25858]	2.2	NC
42	29/5.01	Cytosolic triose-phosphate isomerase	23/10	43.70	27/5.39	CAB75902.1	2.1	NC
46	27/5.18	Protein F18O14.33 [imported]	4/3	8.86	50/6.81	D86328	1.8	NC
53	18/6.58	Hypothetical protein At2g26210 [imported]	6/2	35.56	9.2/6.5	G84657	1.9	NC
26	41/6.79	Peroxidase	65/23	41.76	39/6.21	CAA66959.1	0.3	NC
38	32/6.64	Adenylate kinase 1	3/2	13.01	27/6.91	sp[O82514]	p	NC
39	31/6.62	Adenylate kinase 1	3/2	14.63	27/6.91	sp[O82514]	p	NC
40	31/6.63	Adenylate kinase 1	9/4	27.24	27/6.91	sp[O82514]	p	NC
		**Group III**						
12	58/4.56	Probable ubiquitin-like protein [imported]	2/1	2.90	58/4.81	C84549	NC	2.0
74	43/5.83	Alpha-galactosidase-like protin	3/3	10.49	46/6.19	CAC08338.1	NC	2.1
82	31/5.78	Hydroxyacylglutathione hydrolase cytoplasmic (glyoxalase II)	5/3	24.81	29/5.93	sp[O24496]	NC	1.7
36	36/5.48	Annexin 2	2/1	46.34	4.7/4.8	BAD94993.1	NC	2.4

a) Index in the reference gel. b) Observed Mr and *pI *which were estimated by the electrophoresis mobilities. c) Expected Mr and *pI *which were calculated on the complete sequences. d) Peptides had been identified, the number before solidus represent amount of the peptides had been identified containing overlaps and the number behind solidus represent amount of the peptides excluding overlaps. e) Percentage that the mass of the peptides we identified account for the percentage of total mass of the protein. f) Accession number in NCBI, SWISS-Port, or PIR. g) H/S, ratio of amount of identified protein of the horizontal clinorotation treated samples and the stationary control. NC, no change; p, position of identified protein in gel changed after treatment; H, horizontal clinorotation; S, 1 g stationary control. Group I, proteins showed altered expression in both WT and *pin2 *under the horizontal clinorotation in comparison with stationary controls. Group II, expression of proteins was modified in only WT but apparently unchanged in *pin2 *under the horizontal clinorotation. Group III, expression of proteins did not changed in WT roots, but significantly up-regulated in *pin2 *roots subjected to the horizontal clinorotated treatment.

**Table 2:** Identified proteins whose expression levels changed between *Arabidopsis thaliana *wild-type and *pin *2 root tips exposed to hyper-gravity force (7 g) compared to 1 g control using two-dimensional electrophoresis and liquid chromatography-ion trap mass spectrometry

Spot No^a)^	Exp.^b)^ Mr/pI	Identity of the proteins	**No**. Mat^d)^	% Sq Cov^e)^	Theo.^c)^ Mr/pI	**Acces**. no.^f)^	G/S ^g)^	
							WT	*pin*2
		**Group I**						
9	65/5.66	TCP-1 chaperonin-like protein	14/10	23.52	59/5.97	CAC01806.1	2.6	2.2
22	54/6.00	ATP synthase beta chain 2, mitochondrial	47/21	53.42	60/6.18	sp[P83484]	1.5	1.9
23	44/6.12	Mevalonate diphosphate decarboxylase	3/2	8.98	46/6.33	CAA76803.1	1.6	2.0
30	45/5.72	Isocitrate dehydrogenase	60/20	50.24	46/6.33	CAD24782.1	1.5	1.9
19	54/5.76	Aldehyde dehydrogenase (NAD^+^)-like protein	4/4	12.08	58/7.11	CAB41139.1	2.5	0.5
36	36/5.48	Annexin 2	2/1	46.34	4.7/4.8	BAD94993.1	1.8	3.1
21	54/5.84	Mitochondrial ATP synthase alpha chain	89/20	42.60	55/6.23	sp[P92549]	0.4	0.5
2	73/4.95	Heat shock cognate 70 kDa protein 1	48/21	42.24	71/5.03	sp[P22953]	8.0	15
56	73/5.77	Heat shock cognate 70 kDa protein 1	19/11	24.88	71/5.03	sp[P22953]	13.0	5.7
3	74/5.02	Heat shock cognate 70 kDa protein 3	54/23	42.99	71/4.97	sp[O65719]	2.8	15.0
57	68/5.76	Stress-induced protein sti1-like protein	54/24	46.77	64/6.00	CAB78283.1	4.7	1.6
5	78/5.61	NADH-ubiquinone oxidoreductase 75 kDa subunit, mitochondrial precursor	10/6	13.10	82/6.24	sp [Q 9FGI6 ]	6.4	1.8
38	32/6.64	Adenylate kinase 1	3/2	13.01	27/6.91	sp[O82514]	p	p
39	31/6.62	Adenylate kinase 1	3/2	14.63	27/6.91	sp[O82514]	p	p
40	31/6.63	Adenylate kinase 1	9/4	27.24	27/6.91	sp[O82514]	p	p
		**Group II**						
15	60/5.77	Putative malate oxidoreductase	21/13	33.00	66/6.85	CAB80866.1	2.3	NC
24	44/6.42	Glutathione-dependent formaldehyde dehydrogenase class III ADH	24/9	31.90	41/6.51	CAA57973.1	0.3	NC
		**Group III**						
74	43/5.83	Alpha-galactosidase-like protein	3/3	10.49	46/6.19	CAC08338.1	NC	2.1
82	31/5.78	Hydroxyacylglutathione hydrolase cytoplasmic (glyoxalase II)	5/3	24.81	29/5.93	sp[O24496]	NC	2.0

a)-f) is as the description in the table 1. g) G/S, ratio of amount of identified protein of the hyper-gravity treated samples and its stationary control. NC, no change; p, position of identified protein in gel changed after treatment; G, hyper-gravity treatment; S, 1 g stationary control. Group I, proteins showed altered expression in both WT and *pin2 *under the hypergravity condition in comparison with the stationary controls. Group II, expression of proteins was modified in only WT but apparently unchanged in *pin2 *under the hypergravity. Group III, expression of proteins did not changed in WT roots, but significantly increased in *pin2 *roots subjected to the hypergravity treatment.

**Figure 5:**
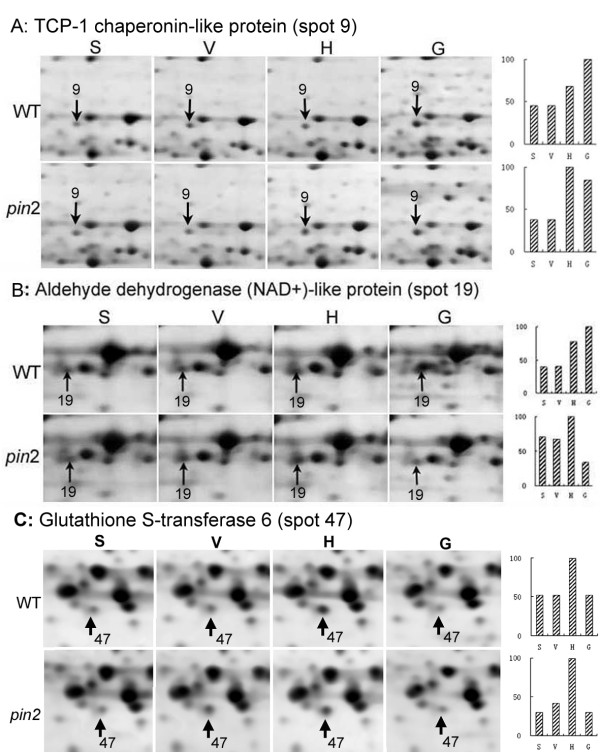
**Examples of Arabidopsis proteins that exhibited similar changes in expression in wild type (WT) and *pin2 *mutant (*pin2*) roots subjected to either horizontal or vertical clinorotation, or hypergravity compared with control roots grown under stationary conditions**. Differentially accumulated protein spots are indicated by arrows. The level of increase or decrease found for each spot is shown in bars on the right. Detailed information for proteins shown in A, B and C is provided in Tables 1 and 2 (spot nos. 9, 19 and 47). The highest level found among various treatments is given as 100. S, stationary control; V, vertical clinorotation; H, horizontal clinorotation; G, hypergravity.

*Group I *proteins showed altered expression in both WT and *pin2 *under the H and/or the G conditions. Eight spots from the H samples (Table 1) and 15 spots from the G samples (Table 2) were part of this group. They included 5 protein spots (nos.9, 22, 23, 30 and 19), whose expression is apparently modified in response to both the H and the G treatment, 3 spots (nos.16, 24 and 47) which exclusively responded to the H treatment, and 10 spots (nos. 2, 3, 5, 21, 36, 38, 39, 40, 56 and 57) which only affected by the G treatment. For example, spot 9 in both WT and *pin2 *roots had a similar expression tendency in response to both the H and the G treatments (Figure 5A). Expression of spot 47 in both WT and *pin2 *apparently increased under the H condition, but was unchanged in the G treatment samples (Figure 5C). In addition, expression of spot 19 in WT and *pin2 *roots was up-regulated by the H treatment, but under the G treatment, its expression was up-regulated in WT, and down-regulated in *pin2 *roots (Figure 5B).

**Figure 6:**
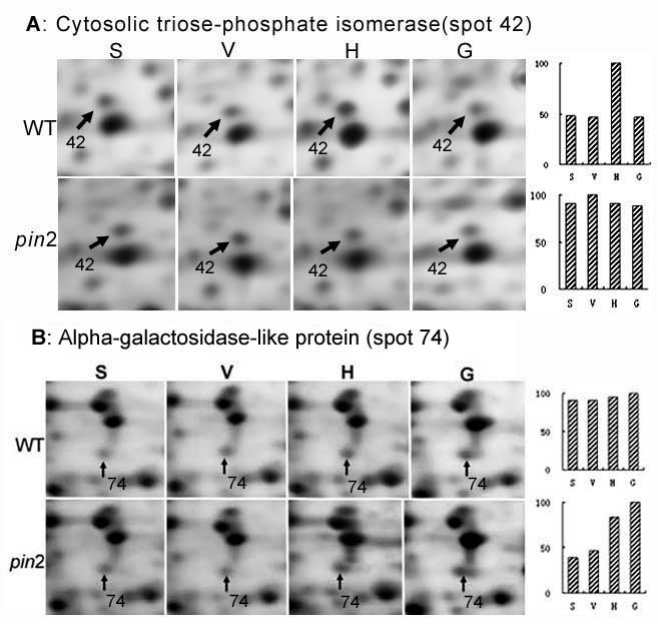
**Examples of Arabidopsis proteins that exhibited a different pattern of expression between wild type (WT) and *pin2 *mutant (*pin2*) roots**. A, example of a protein that changed little in *pin2 *roots under the horizontal clinostat rotation condition, but changed significantly in WT roots. B, a protein that changed little in WT roots, but increased significantly in *pin2 *roots exposed to either horizontal clinostat rotation or to hypergravity conditions. Detailed information for proteins shown in A and B are provided in Table 1 and 2 (spots 42 and 74). S, stationary control; H, horizontal clinorotation; V, vertical clinorotation; G, hypergravity.

*Group II *comprises protein spots with modified expression in WT but apparently unchanged expression in *pin2 *in response to the H or the G treatment. In *pin2 *roots, 10 spots (nos. 20, 21, 25, 26, 38, 39, 40, 42, 46 and 53) were insensitive to the H condition, but sensitive to the G treatment; spot 24 exhibited an elevated expression level under the H condition but was insensitive to the G treatment, whereas spot 15 was unchanged under both the H and the G conditions (Tables 1 and 2). All these proteins in WT roots are responsive to the H and/or the G treatments, such as spot 42, whose expression level showed little change in *pin2*, but was significantly up-regulated in WT roots subjected to the H clinostat rotation condition (Figure 6A).

*Group III *is represented by four spots. Their expression did not change in WT roots subjected to the H and/or the G treatment but was significantly up-regulated in *pin2 *roots (Tables 1 and 2). In WT roots, two spots (nos. 12 and 36) were not significantly changed under the H condition, but increased under the G condition, while the other two spots (nos.74 and 82) were insensitive to both the H and the G treatments. In contrast, the expression of all these protein spots in *pin2 *roots was affected by the H and/or the G treatment (*i.e *spot 74 in Figure 6B).

**Figure 7:**
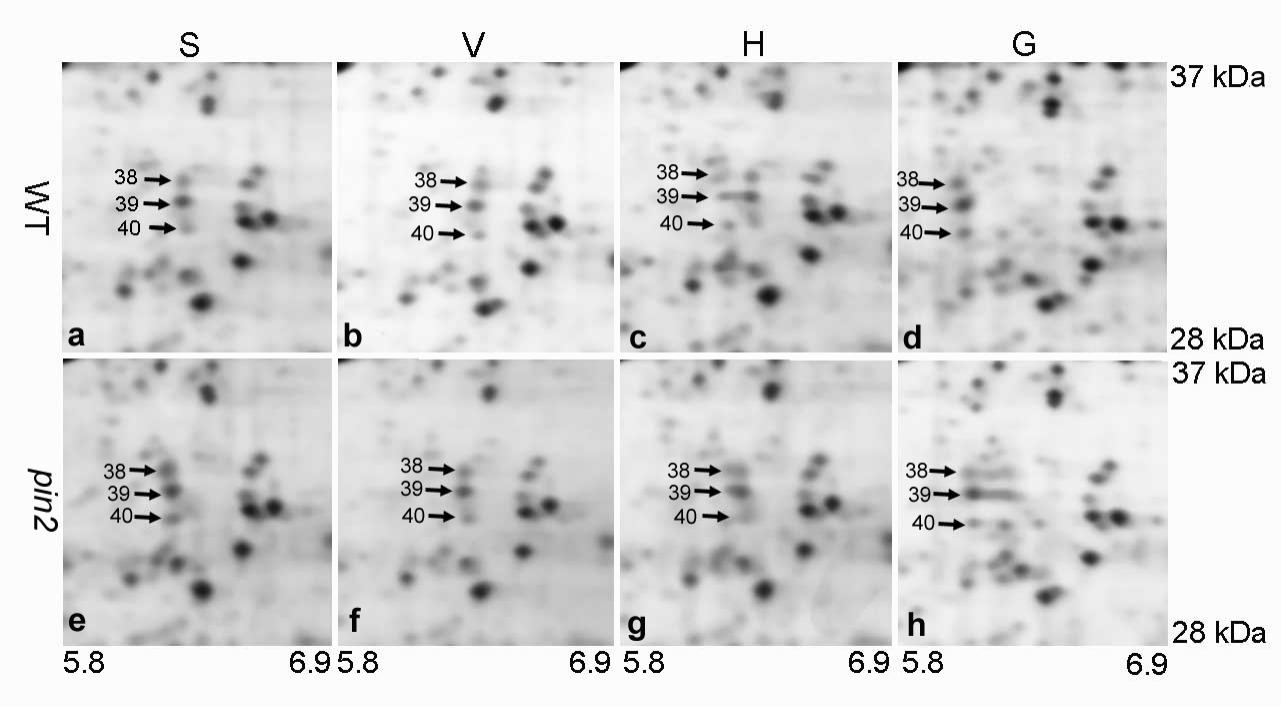
**Details of silver-stained 2-D gels of proteins whose position changed in wild type (WT) and *pin2 *mutant (*pin2*) roots subjected to either horizontal or vertical clinorotation or hypergravity treatments compared with control roots grown under the stationary condition**. The proteins (spot nos. 38, 39, 40) indicated by arrows were identified as the same protein, adenylate kinase 1. They migrated towards more acid regions after the horizontal clinorotation and hypergravity treatment. The ranges of pI and molecular masses (kDa) of the marked proteins circumscriptions are indicated. S, stationary; H, horizontal clinorotation; V, vertical clinorotation; G, hypergravity.

**Figure 8:**
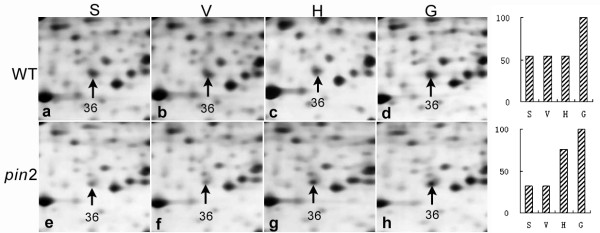
**The magnified regions of 2-D images showing the expression pattern of spot 36 in the WT and *pin2 *roots subjected to horizontal clinorotation (H) or hypergravity (G) in comparison with stationary (S) or vertical clinorotational (V) controls**.

Among the gravity-responsive proteins identified in this study, some fall into one group in response to one type of treatment and into another group when subjected to a different treatment. For example, spots 38, 39 and 40 belong to group II in the H samples and to the group I in the G samples (Tables 1 and 2; Figure 7). In H samples, spot 36 is a member of group III, but belongs to group I in the G samples (Tables 1 and 2; Figure 8). PIN2 is important for the basipetal transport of auxin in roots, which is important to signal the root elongation zones of gravity signals perceived in the root cap. In the absence of PIN2, auxin is retained in the root tip (Additional file 1 Figure S4), thereby perturbing the flow of auxin from the root tip to the elongation zone [18,43]. We assume that the proteins, with similar expression patterns in both *pin2 *and WT roots under the H and/or the G conditions, may correspond to proteins involved in general stress responses, or in PIN2-independent gravity responses. In turn, the proteins in *pin2 *roots that behave differently from those in WT under altered gravitational conditions might correspond to proteins involved in potential pathways/processes related to PIN2 function.

### Group I-type proteins may reflect a general stress response and a PIN2-indenpendent gravity response of roots subjected to an altered gravitational forces

Tables 1 and 2 demonstrate that five protein spots are representative of group I-type samples whose expression in both WT and *pin2 *roots was apparently modified in response to both the H and the G treatment. These spots correspond to t-complex peptide-1 (TCP-1) chaperonin-like protein, mitochondrial ATP synthase beta chain2, mevalonate diphosphate decarboxylase (MVD), isocitrate dehydrogenase (IDH) and NAD^+^-aldehyde dehydrogenase (ALDH)-like protein. TCP-1 chaperonin has been reported to be associated with microtubular structures and to function in microtubule-driven transport of vesicles [44,45]. The up-regulation of TCP-1 chaperonin-like protein in both WT and *pin *2 roots under both H and G condition in this study might indicate the involvement of the cytoskeleton in the response to an altered gravitational force. IDH and β-chain 2 of the ATP synthase complex are likely to regulate flux restrictions in the tricarboxylic acid cycle (TCA) and electron transport, thereby increasing the synthesis of ATP, which can supply extra energy for cells to counteract the stress under altered gravity conditions. MVD is a key enzyme in the isoprenoid and sterol biosynthetic pathway, which takes part in diverse cellular functions, such as respiration, protein glycosilation and signal transduction [46]. ALDHs have been considered to play a major role in the detoxification of aldehydes, which are generated in plants exposed to abiotic stress [47]. The similar behaviors of these proteins in the *pin2 *mutant and its WT roots in response to the altered gravity may suggest that mutation in PIN2 may not affect a common stress response of roots. Clinorotation probably results in the functional compensation of the directional component of gravity, which can cause the statolith to move away from the distal pole of the statocyte [48], whereas hypergravity can potentiate the gravity response in plant roots [49]. Both of these two types of altered gravity stimuli are suggested to be acting as a significant stressor to plants [42,50]. Thus, the common response proteins in group I appear to correspond to proteins modified by general stress associated with changes in the gravity environment.

In addition to those proteins that exhibited common response after the H and the G treatment, we also found three group I proteins that responded specifically to randomized gravity signals (glutathione-dependent formaldehyde dehydrogenase, putative inosine-5'-monophosphate dehydrogenase and glutathione S-transferase 6) and ten spots, identified as seven proteins whose expression was modified exclusively in the G treated samples (heat shock cognate 70 kDa protein 1, heat shock cognate 70 kDa protein 3, NADH-ubiquinone oxidoreductase75 kDa subunit, mitochondrial ATP synthase alpha chain, annexin and adeylate kinase 1). These data suggest that different types of gravity stimulation might activate different types of response pathways. For example, glutathione-dependent formaldehyde dehydrogenase (GS-FDH), putative inosine-5'-monophosphate dehydrogenase (IMP-DH) and glutathione S-transferase (GST) 6, have been suggested to play a major role in detoxification processes in plants exposed to abiotic stress. They have also shown previously to be up-regulated by clinorotaion in Arabidopsis culture cells [40]. The cDNAs of GS-FDH and GST in Arabidopsis root apex cells were shown to be up-regulated by altered gravity and mechanical stimulation in previous studies [33,36]. In this study, the expression levels of GS-FDH, putative IMP-DH, and GST6 in both WT and *pin2 *roots specifically modified by the H treatment suggest that there might exist a group of proteins that are sensitive to randomized gravity but not to hypergravity. In addition, the absence of PIN2 might not affect the response of these proteins to clinorotation-type stress.

The expression of heat shock cognate 70 kDa (HSP 70) protein 1(spot 2 and spot 56), HSP70 protein 3(spot 3), NADH- ubiquinone oxidoreductase (UBOR) 75 kDa (spot 5) and stress-induced protein stil-like proteins (spot 57) were apparently modified only by hypergravity, and not by clinostat treatment. Consequently these proteins might represent those sensitive to the intensity of gravitational forces and not be dependent on functional PIN2 proteins. For example, during early stages of the gravity signal transduction pathway [51,52], J-domain proteins have been shown to interact with the HSP70 molecular chaperone in root columella cells [53,54]. Together, these results indicate that a subset of *Group I *proteins are involved in general stress response and in PIN2-independent early phase gravity signal transduction pathways.

### Group II and III-type proteins may be involved in auxin-mediated steps of gravity signaling in root tips

Of the eleven group II protein spots of WT plants subjected to the H treatment that exhibited changes in protein levels/pI (Table 1), three (spot nos 38, 39 and 40 in Figure 3 and Figure 7) were identified as adenylate kinase 1, with altered pIs and seven proteins exhibited an increase in expression levels. These seven proteins were identified as enolase, glyceraldehyde-3-phosphate dehydrogenase(GAPDH), cytosolic triose-phosphate isomerase (TPI), putative malate oxidoreductase, mitochondrial ATP synthase α-chain, and two other proteins with function unknown (spots 46 and 53 in Figure 3 and Table 1). Among these proteins, enolase, GAPDH and TPI are key enzymes of the glycolysis pathway, while MOR and alpha chain of ATP synthase are key mitochondrial enzymes associated with the TCA and oxidative phosphorylation pathways, respectively. This result might be related to that the increase in glucose and fructose in WT roots subject to H and G conditions (Figure 2a). In *pin *2 roots, the pool size of glucose was also up-regulated by the H treatment, but the content of fructose did not change under either H or G conditions (Figure 2b). Right now, we cannot explain this observation. But obviously, no sucrose hydrolysis is involved in the hexose production, because this would yield identical changes for both hexoses. It has been reported that plant cells harbor sugar-sensing and -signaling systems that regulate gene expression and control the metabolic processes needed for growth and signaling. In this context, fructose has been proposed to be involved in a signaling pathway that interacts with absicic acid-ethylene-signaling pathways [55]. At present we do not know if there is an interaction between the fructose-specific signaling pathway and auxin.

Our finding that altered gravity conditions affect the fructose pool size as well as key enzymes of the glycosis and the TCA pathways in both *pin2 *and WT roots provides new evidence in support of that fructose might be involved in the auxin-mediated gravity signaling pathway. Furthermore, there is now increasing evidence that enzymes known as "bifunctional" or "moonlighting"enzymes can have more than one function [56]. For example, GAPDH also has a role of mediating reactive oxygen species (ROS) signaling in plant cells [57]. Altered expression and regulation of alpha-chain of mitochondrial ATP synthase genes in response to ROS changes are well-documented in animal cells [58,59]. For these reasons, the group II enzymes we have identified (enolase, GAPDH and TPI, MOR and ATP synthase alpha chain) could also be involved in auxin-mediated later phase gravity signaling, including a ROS mediated physiological response. This idea is strengthened by the observation on spot 26, a peroxidase, which is significantly down-regulated (3.3-fold) in WT root tip cells, while remaining unchanged in *pin *2 roots. Peroxidase is known to mediate the generation of ROS, which may function as a downstream component in auxin-mediated signal transduction in root gravitropism [60,61]. How auxin and ROS are integrated into a physiological gravity signaling pathway remains to be determined.

Three spots (nos. 38, 39 and 40 in Figure 3 and 7) were identified as adenylate kinase 1 with molecular weights 31-32kD. Their pIs ranged from 6.62-6.64 in the stationary control, and migrated towards the more acid region (pI 6.34) in WT when subjected to the H treatment. No pI changes occurred in the horizontally clinorotated *pin2 *mutant root tips relative to the S or V controls (Figure 7a-c and 7e-g). The pI of this enzyme became even more acid (pI 6.15) under the hypergravity conditions in both WT and *pin2 *roots (Figure 7d and 7h). Database screening revealed that the genome of Arabidopsis contains 10 genes with adenylate/cytidylate kinase, seven of which have been identified as putative adenylate kinases [62]. In mammalian tissues, adenylate kinase 1 localizes to the cytoplasm and is highly expressed in brain, testis and cardiac cells [63]. There are only a few reports on specific adenylate kinase isoforms in plants. Carrari et al. (2005) found that in Arabidopsis a knockout mutant defective in AMK1 had a phenotype with decreased adenylate, elevated amino acid levels, and reduced starch content in roots [64]. The cDNA clone of Arabidopsis adenylate kinase 1 was isolated and was predicted to encode a protein with molecular mass of 27 kDa and pI of 6.91. In the present study, the difference in molecular mass and pI among protein spots 38, 39 and 40 indicate that they might be post-translationally modified (e.g. phosphorylation). Stress-induced shifts in pI of proteins has been reported previously [65,66] and transient changes in molecular weight of specific proteins have been noted in Arabidopsis roots [38]. This is the first study to report a shift of pI of adenylate kinase 1 induced by altered gravity. It is possible that the changes observed in adenylate kinase1 pI might be related to the gravistimulation response of adenosine kinase reported by others [67]. However, the reason for the insensitivity of this enzyme in *pin 2 *subjected to the H rotation is unclear. Thus, we postulate that the adenylate kinase 1 changes may be associated with an auxin-mediated early phase gravity signaling event in root cap cells.

An alpha-galactosidase-like protein and hydroxyl acylglutathione hydrolase were significantly increased in *pin2 *but not in WT roots exposed to both H and G (Table 1). The probable ubiquitin-like protein and annexin (ANN) 2 also exhibited different responses in *pin2 *and in WT roots under the H condition, while demonstrating similar expression patterns in both *pin2 *and WT roots subjected to the G condition (Tables 1 and 2). We suggest that mutation of the PIN2 in roots could result in altered sensitivities of some proteins in response to changes in the gravitational environment. For example, the abundance of ANN 2 in *pin2 *mutant root tips was 61% of that in WT samples under the S or V control condition (Figure 8a, b, e and 8f). However, its abundance was significantly increased in *pin2 *roots subjected to the H treatment and approached the control level of WT roots (Figure 8c and 8g). Up-regulation of ANN 2 was observed in both WT (1.8-fold) and in *pin 2 *mutant (3.1-fold) root tips under the G condition (Figure 8d and 8h) compared to S or V controls.

**Figure 9:**
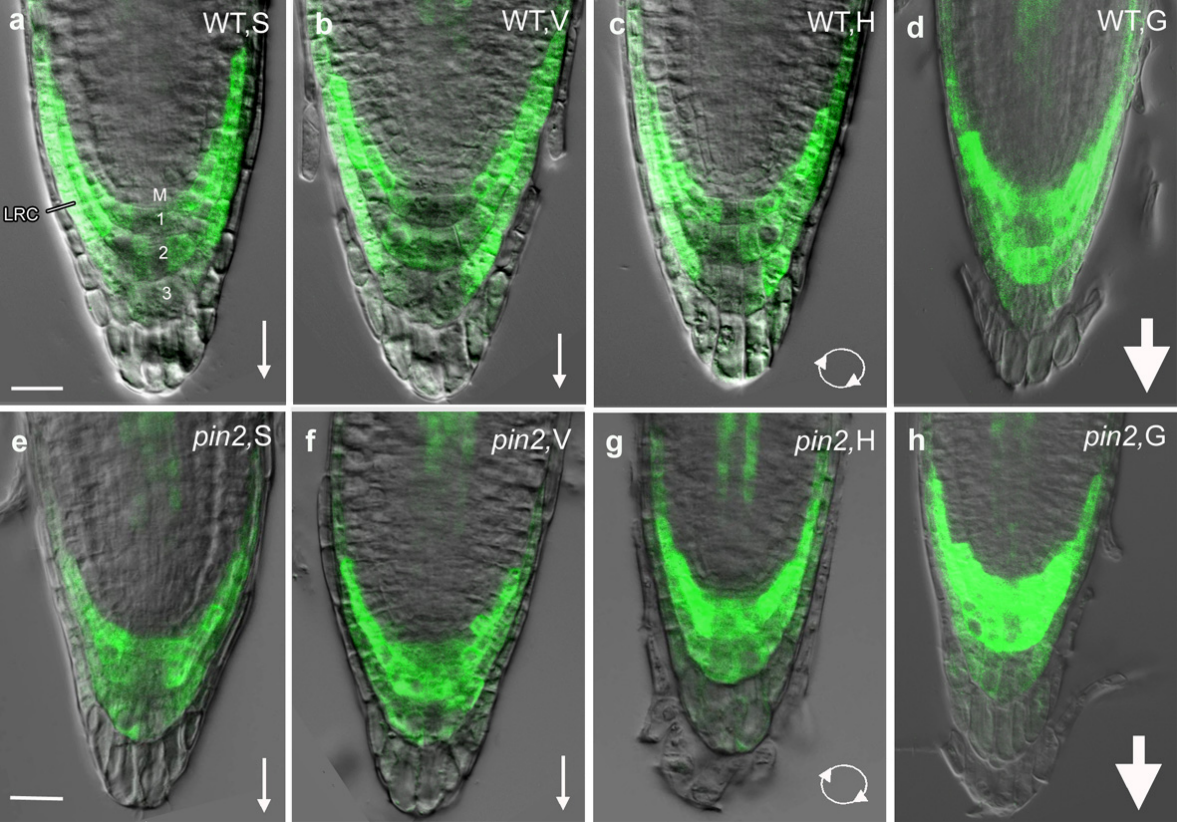
**Expression of the ANN2::green fluorescent protein (GFP) fusion in root tips of wild type (WT) and *pin*2 mutant background seedlings**. Longitudinal opitical sections through root tips of 6-day old WT (a-d) and *pin*2 (e-h) seedlings subjected to the horizontal clinorotation(H) or hypergravity (G) condition in comparison with its controls under 1 g stationary control (S) or the vertical clinorotation (V) conditions. a, b and c, Representative images of WT root tips depicting a meristematic columella initial cell (M), and three stories of derived columella cells (nos. 1,2 and 3). Most of the GFP signal was confined to the lateral root cap (LRC) cells and to a lesser extent to the columella cell region in plants grown under the S, V and H conditions, respectively. d, Representative images of ANN2::GFP expression in wild-type roots exposed to 7 g hypergravity treatment. Note that a strong GFP signal is seen both in columella cells and lateral root cap cells. e and f, Representative images of ANN2::GFP expression in *pin2 *roots under the control conditions (S and V). g and h, Representative images of *pin2 *root tips under H and G condition, respectively. Note the increase in GFP signal in the first and the second tier columella cells. Arrows indicate direction of gravity. Bars = 20 μm.

To further investigate if the absence of PIN2 could be responsible for different protein behaviors between WT and *pin2 *mutant root tips under altered gravitational conditions, we generated a *ANN2pro:: ANN2-GFP *fusion construct with the native promoter and analyzed its expression in transformed Arabidopsis seedlings in both WT and *pin2 *mutant backgrounds by confocal fluorescence microscopy (Figure 9). In WT plants expressing the *ANN2pro::ANN2-GFP *construct, ANN2 was mainly restricted to lateral root-cap cells and to a lesser extent in the columella region under the S and V conditions (Figure 9a and 9b). In contrast, prominent ANN2-GFP signals were found in the columella cells of *pin2 ANN2pro::ANN2-GFP *plants under S and V conditions (Figure 9 e and f compared with Figure 9a and 9b, respectively). An increase in ANN2-GFP signals was also seen in the columella cells of *pin2 ANN2pro::ANN2-GFP *plants subjected to H treatment (Figure 9g). In contrast, no increase has observed in WT plants (Figure 9c). A striking accumulation of ANN2-GFP was produced in columella cells and in lateral root cap cells of both WT and *pin2 *root tips subjected to hypergravity (Figure 9d and 9h). This observation is consistent with our proteomic analysis on spot 36 (Figure 8), and indicates that of PIN2 might be required for proper expression and localization of ANN2 in roots in response to changes of gravitational conditions. In addition, altered gravity mediate differential distribution of ANN2 in lateral root cap and columella cells of both WT and *pin2 *roots indicated the possible involvement of PIN2 in regulating the passage of information from columella cells to lateral root cap cells in the gravity signal transduction pathway, even though no difference was observed in the distribution of ANN2 between S and H treated in WT roots.

## Conclusions

In this study, we have identified 25 proteins whose expression level/pI were altered by clinorotation and/or hypergravity force in Arabidopsis WT and/or *pin*2 mutant roots. As such it complements other studies in which the mechanism of gravitropism and gravity signal transduction/transmission have been investigated by means of DNA microarray, suppression subtractive hybridization, and proteomics [34, 36, 38, 39 and 40]. The most interesting observation of this study is that three distinct patterns of protein expression were found in WT and *pin*2 mutant roots subjected to different gravity treatments.

The first class consisted of proteins whose expression was significantly elevated in both WT and *pin2 *mutant roots in response to altered gravity conditions. These proteins were involved in detoxification (GST 6, NAD^+^-ADH, GS-FDH), in chaperone functions(HSP70) and in mediating resistance to stress factors correlated with energy metabolism (mitochondrial ATP synthase, putative inosine-5'-monophosphate dehydrogenase, isocitrate dehydrogenase). Surprisingly, some proteins, such as cytosolic triose phosphate isomerase, glyceraldehydes-3-phosphate dehydrogenase, enolase, and putative malate oxidoreductase, which are key enzymes in glycolysis or linked directly to the reduction of NAD^+ ^to NADH, became insensitive to altered gravity in *pin2 *roots. The finding that the key enzymes of glycolysis as well as gravity-responsive proteins became insensitive to altered gravity in *pin2 *mutant roots adds to the interest in PIN2 protein as a modulator of responses of roots to gravity.

PIN2 is important for the basipetal transport of auxin in roots, which plays a critical role in the transmission of gravity signals perceived in the root cap to the cells in the elongation zone. For this reason, the observed differences in protein expression between WT and *pin2 *mutant root tips have the potential of identifying important proteins involved in the gravity signaling transmission pathway. For example, HSP 70, ADK1 and ANN2 may be involved in early phases of gravity signal transduction, and the changes in HSP 70 proteins in response to gravitational treatments can be rationalized in view of the reported contribution of J-domain proteins in early phases of gravity signal transduction [53,54]. The pIs of three adenylate kinase spots were shown to change substantially in horizontally clino-rotated and hyper-gravity stimulated samples relative to controls, suggesting posttranslational regulation during an early phase of gravity transduction. The ANN2 responses observed in this work might have something to do with early responses of roots to altered gravitational stimulation. This idea is strengthened by the observation of a differential increase of ANN2-GFP signals in the columella cells of WT and *pin2 *plants subject to the H or G treatment.

In an earlier study, transcriptional profiling demonstrated that about 1.7% of the 8,300 genes studied exhibited significant expression changes within the first 30 min of gravity stimulation [33]. Since each gene has the potential of giving rise to multiple proteins by means of alternative splicing or post transcriptional modulation, the number of proteins involved in the regulatory networks for gravitropic control could be significantly higher. The proteins identified in this investigation represent only a small part of the Arabidopsis proteome, and many other gravity-responsive proteins will likely be identified in the future. For example, proteins associated with the actin cytoskeleton are known to contribute to responses to different gravity environments [68,69]. Thus, deeper proteomic analyses have the potential of yielding further insights into the molecular details of the gravity response pathways in Arabidopsis.

## Methods

### Plant materials

Seedlings from *Arabidopsis thaliana *ecotype Columbia (Col-0) wild-type and an agravitropic *pin2 *mutant were used for the experiments. Seeds of *pin 2 *mutant and DR5::GUS wild-type were kindly provided by Dr. Palme (University of Freiburg Germany). Seeds were surface-sterilized for 20 min with 2% (v/v) sodium hypochlorite solution containing 0.01%(v/v) Triton X-100. They were rinsed five times with sterile distilled water and sown on Whatman No 2 filter paper. The paper was covered with Murashige and Skoog (MS) media [70] supplemented with 0.8%(w/v) agar and 1%(w/v) sucrose (about 20 seeds on a filter 5 × 5 cm^2^). The filter paper with MS media was placed in a tissue culture flask (200-mL flask; 6.5 × 6.5 × 10 cm^3^; Jiafeng Co., Ltd., Shanghai, China) and stored at 4°C for 2 days. Plants were grown vertically in the tissue culture flasks (Additional file 1 Figure S1) at 22°C for 3 days under 18 h of illumination (about 600 μEm^-2^s^-1^), then seedlings were kept in the dark for 24 h prior to the altered gravity treatments.

### Clinostat and hypergravity treatments

For clinostat treatment, a one-axis 1-π clinostat facility (SB-01) was used as described [40]. Briefly, the clinorotation treatment in this study consisted of a clinostat oriented horizontally (H) and rotating at 5 rpm (e.g. the level of acceleration applied to the seedlings was less than 7 × 10^-4^g at a radius of 25 mm). In addition to the stationary 1 g control (S), a vertically oriented clinostat (V) rotating at 5 rpm was used as a control to evaluate potential mechano-stimulation artifacts from the clinostat motor and the side-effects of rotation itself. To this end, 6-day-old Arabidopsis seedlings obtained in control conditions were placed on the clinostat described above for 12 h (Additional file 1 Figure S1). For hypergravity(G) treatment, the seedlings at the same growth stage as those in clinostat treatments were selected and submitted to a hypergravity treatment by centrifugation (Beckman, J2-HS) at 7 g for 30 min (Additional file 1 Figure S1). All the treatments were performed in total darkness. The root-tip region with a length of 5-10 mm was excised and frozen in liquid nitrogen immediately. All samples were stored at -80°C prior to experiment.

### Carbohydrate analysis

The soluble sugar and starch content were determined as previously described [40]. Briefly, about 10 mg of the root tips were ground in liquid nitrogen to powder and incubated in 80%(v/v) ethanol at 60°C for 1 h, dried, and extracted in 200 μL ddH_2_O at room temperature for 15 min with occasional vortexing, followed by centrifugation at 10 000 g for 10 min. The supernatants were used for soluble sugar assays and the residual pellets used for starch assay. The absorbance was recorded on a microplate reader system (Sunrise Tecan, USA). At least five independent samples were measured to obtain the mean values.

### Proteins extraction

The root tip proteins were extracted using a modified trichloroacetic acid/acetone procedure as described previously [40]. The root tissues were ground in liquid nitrogen to a fine powder and resuspended in an ice-cold solution of 10% (w/v) trichloroacetic acid(TCA) in acetone with 0.07% (w/v) DTT and centrifuged for 30 min at 35 000 *g*. The pellets were lyophilized and solubilized in lysis buffer (7 mol L^-1 ^urea, 2 mol L^-1 ^thiourea, 4%(v/v) CA-630, 32 mmol L^-1 ^Tris-HCl pH 6.8, 1 mmol L^-1 ^PMSF, 14 mmol L^-1 ^DTT, and 0.2%(v/v) Triton X-100) and then centrifuged at 12 000 *g *for 15 min. The proteins in the supernatant were precipitated by adding four volumes of ice-cold acetone and centrifuged at 12 000 *g *for 15 min. The purified pellets were dissolved in rehydration buffer containing 8 mol L^-1 ^urea, 2%(w/v) CHAPS, 18 mmol L^-1 ^DTT, 0.5% (w/v)IPG buffer, pH 3-10 and a trace of bromophenol blue. The protein concentrations were quantified using the Bradford method [71]. Four samples per treatment (H, V, S or G) were prepared from individual experiments of WT and *pin2 *seedlings. All samples were stored at -80°C prior to electrophoresis.

### Gel electrophoresis

2-DE was performed primarily according to Yu et al. (2001)[72]. 100 μg and 800 μg of total proteins were loaded onto analytical and preparative gels, respectively. Using an IPG strip gel (IPG strip; pH 3-10 no linear, 13 cm, Pharmacia), the first dimensional isoelectric focusing (IEF) was performed at low voltages (500-1000 V) during the first 2 h and then continued with a maximum setting of 8 000 V to reach a total of 80 kVh. After IEF separation, the gel strips were equilibrated for 2 × 15 min in an equilibration buffer containing 6 mol L^-1 ^urea, 30%(w/v) glycerol, 2%(w/v) SDS, 50 mmol L^-1 ^Tris-HCl buffer (pH 8.8). 1% (w/v) DTT was added to the first equilibration buffer and in the second equilibration buffer DTT was replaced with 2.5% (w/v) iodoacetamide. The second dimensional SDS-PAGE (2-DE) was carried out exactly as described previously [40]. After 2-DE, protein spots in analytical gels were visualized by silver nitrate staining [72], and the preparative gels were stained with Colloidal Coomassie Brilliant blue G-250. Eight analytical gels for each sample per treatment (H, V, S or G), resulting from duplicate runs of individual samples, were completed.

### Image acquisition and analysis

The silver and Coomassie blue-stained 2-D gels were scanned at an optical resolution of 84.7 μm/pixel using a GS-710 imaging densitometer (Bio-Rad). Spot detection and matching were performed using ImageMaster 2D Platinum software (GE Healthcare) to confirm that the protein spots to be excised from Coomassie blue-stained gels were corresponding to the same spots in silver-stained gels. A matchset consisting of 32 images of WT or *pin 2 *gels, eight for the H samples, eight for the V samples, eight for the G samples, and eight for the S samples, was created, and one image from S was selected as the matchset standard for spot matching. Only those with significant and reproducible changes were considered to be differentially accumulated proteins. The abundance of each protein spot was estimated by the percentage volume. For example, the individual spot volumes were normalized by dividing their optical density (OD) values by the total OD values of all the spots present in the gel, and expressed as % Vol. The significance of expression differences of protein spots between treatments (H, V, or G) and stationary control (S) was estimated by Student's *t*-test, P < 0.05.

### Liquid chromatography-ion trap-mass spectrometry (LC-IT-MS) and protein identification

In-gel digestion, LC-IT-MS and protein identification were performed according to the methods described previously [40]. Briefly, protein identification using MS/MS raw data was performed with the SEQUEST software (University of Washington, licensed to Thermo Finnigan) searching program against the NCBI Arabidopsis database (http://www.ncbi.nlm.nih.gov).

### ANN2pro::ANN2:eGFP plasmid construction and stable transformation of Arabidopsis

The coding sequence (CDS) of *ANNEXIN2*(*ANN2*) without stop codon was amplified by PCR from a Col-0 cDNA using the primers with the restriction sites underlined 5'-ATCGGATCCATGGCGTCTCTCAAAGTCCC-3' and 5' -CGTACTAGTAGCATCGCCATGTCCGAGA-3' and ligated into a pBluescript SK vector that contained eGFP. A 1150 bp DNA fragment upstream from ANN2 start codon corresponding to the putative promoter was also amplified by PCR with the primers 5'-ATCGTCGACTTTTTATTTTTCTTACGCGCATTG-3' and 5'- CGTGGATCCTGTTGGGATTAGTCTTTAAC-3' and cloned into the ANN2:eGFP containing pBluescript SK vector, resulting in *ANN2pro:: ANN2:eGFP*, which was then cleaved and ligated into the Cambia 1301 vector. Transformation of Arabidopsis plants (WT Col-0 and *pin2 *mutant) through the *Agrobacterium tumefaciens *strain GV3101 was performed by the floral dip method according to the methods of Clough and Bent (1998)[73].

## List of abbreviations

LC-IT-MS: liquid chromatography-ion trap-mass spectrometry; MS/MS: the tandem mass; pI: isoelectric; Mw: molecular weight; GFP: green florescent protein; H: horizontal clinostat rotation; V: vertical clinostat rotation; S: 1 g stationary condtion; G: hypergravity; WT: wild type; ANN: annexin; TCP: chaperonin-like protein; MVD: mevalonate diphosphate decarboxylase; IDH: isocitrate dehydrogenase; ALDH: aldehyde dehydrogenase; GS-FDH: glutathione-dependent formaldehyde dehydrogenas; IMP-DH: inosine-5'-monophosphate dehydrogenase; GST: glutathione S-transferase; HSP: heat shock cognate; UBOR: ubiquinone oxidoreductase; GAPDH: glyceraldehydes-3-phosphate dehydrogenase; TPI: cytosolic triose-phosphate isomerase; TCA: tricarboxylic acid cycle; ROS: reactive oxygen species.

## Competing interests

The authors declare that they have no competing interests.

## Authors' contributions

CT carried out the protein function studies and participated in the proteomic analysis. HW carried out the proteomic analysis. YZ participated in the proteomic analysis. BQ carried out gravity response studies. GX participated in fluorescent microscopy analysis. HZ conceived of the study, participated in its design and coordination and drafted the manuscript. All authors read and approved the final manuscript.

## Supplementary Material

Additional file 1 **information about the influence of the altered gravitational force on gravity response and protein expression of roots of both Arabidopsis thaliana wild-type and *pin2 *mutant**. This file provides information on distribution of protein spots whose intensities were altered by clinorotation or hypergravity treatment in Arabidopsis wild-type and *pin2 *root tips (see Table S1 and Table S2), the experimental design (Figure S1), gravitropic response of roots under different gravitational conditions (Figure S2 and S3) and auxin distribution pattern in wild-type and *pin2 *mutant root tips (Figure S4). Click here for file
