# Supplementary material for: A proteomic approach to analyzing responses of Arabidopsis thaliana root cells to different gravitational conditions using an agravitropic mutant, pin2 and its wild type
Source: Proteome Sci. 2011 Nov 16;9:72. doi: 10.1186/1477-5956-9-72 (PMC3228730; doi:10.1186/1477-5956-9-72)
Supplement: Additional file 1 — information about the influence of the altered gravitational force on gravity response and protein expression of roots of both Arabidopsis thaliana wild-type and pin2 mutant. This file provides information on distribution of protein spots whose intensities were altered by clinorotation or hypergravity treatment in Arabidopsis wild-type and pin2 root tips (see Table S1 and Table S2), the experimental design (Figure S1), gravitropic response of roots under different gravitational conditions (Figure S2 and S3) and auxin distribution pattern in wild-type and pin2 mutant root tips (Figure S4). [file 1477-5956-9-72-S1.PDF]

Additional File1

Table S1. Distribution of protein spots whose intensities were altered by clinorotation or hypergravity treatment in Arabidopsis wild-type root tips.

Spot No	Obs. Mw/ pI	Treatment V	H	G	Spot No	Obs. Mw/ pI	Treatment V	H	G
9	65/5.66	NC	+	+	7	66/5.11	ND	ND	N
15	60/5.77	NC	+	+	8	66/5.28	+	+	NC
19	54/5.76	NC	+	+	10	62/5.77	+	+	+
21	54/6.00	NC	+	-	11	60/4.56	+	+	+
22	54/6.00	NC	+	+	13	54/5.34	-	-	+
23	44/6.12	NC	+	+	14	54/4.84	-	+	+
24	44/6.42	NC	+	-	16	57/6.08	-	+	-
30	45/5.72	NC	+	+	17	63/6.67	+	+	+
38	32/6.64	NC	p	p	18	52/4.50	+	-	-
39	31/6.62	NC	p	p	27	42/5.03	+	+	NC
40	31/6.63	NC	p	p	28	44/5.34	-	+	-
20	55/5.79	NC	+	NC	29	41/5.30	-	-	+
25	41/6.66	NC	+	NC	31	40/5.13	-	NC	-
26	41/6.79	NC	-	NC	32	38/5.96	-	-	+
42	29/5.01	NC	+	NC	33	36/6.80	-	-	+
46	27/5.18	NC	+	NC	34	38/6.90	-	+	-
47	25/5.73	NC	+	NC	35	32/4.74	-	+	-
53	18/6.58	NC	+	NC	37	36/4.79	+	+	+
2	73/4.95	NC	NC	+	41	29/4.85	-	+	+
3	74/5.02	NC	NC	+	43	29/5.13	-	-	+
5	78/5.61	NC	NC	+	44	29/5.17	-	-	-
36	36/5.48	NC	NC	+	45	27/5.19	+	NC	-
56	73/5.77	NC	NC	+	48	25/6.58	-	-	-
57	68/5.76	NC	NC	+	50	22/4.70	-	+	-
1	68/4.92	+	+	+	51	20/4.59	-	+	-
4	77/5.20	-	-	+	52	21/6.12	+	+	-
6	81/5.65	+	-	+	54	31/5.78	+	+	+
49	23/4.71	-	-	-					

The spot No refers to the respective gel. Obs., observed molecular weights (kDa) and isoelectric points, which were estimated from electrophoretic mobilities. +，upregulated expression; -，downregulated expression; NC, no change; N, detected only after treatment; ND, not determined; p, position changed; V, vertical clinorotation; H, horizontal clinorotation; G, hypergravity treatment.

Table S2. Distribution of protein spots whose intensities were altered by clinorotation or hypergravity treatment in Arabidopsis mutant (*pin*2) root tips.

Spot No	Obs.	Treatment			Spot No	Obs.	Treatment		
	pI/Mw	V	H	G		pI/Mw	V	H	G
9	65/5.66	NC	+	+	50	22/4.7	-	+	+
19	54/5.76	NC	+	-	51	20/4.59	+	+	-
22	54/6.00	NC	+	+	52	21/6.12	+	+	+
23	44/6.12	NC	+	+	55	72/4.85	+	+	+
30	45/5.72	NC	+	+	58	71/5.8	ND	ND	N
36	36/5.48	NC	+	+	59	59/5.11	+	+	+
74	44/5.81	NC	+	+	60	61/5.30	-	+	+
82	31/5.78	NC	+	+	61	64/5.46	+	+	+
12	58/4.56	NC	+	NC	62	63/5.50	+	+	+
16	57/6.08	NC	+	NC	63	64/6.73	-	-	-
24	44/6.42	NC	+	NC	64	63/6.76	-	-	-
47	25/5.73	NC	+	NC	65	48/4.90	-	-	+
2	73/4.95	NC	NC	+	66	45/5.09	-	-	-
3	74/5.02	NC	NC	+	67	47/5.32	-	-	-
5	78/5.61	NC	NC	+	68	45/5.39	+	NC	+
21	54/6.00	NC	NC	-	69	51/5.54	+	NC	-
38	32/6.64	NC	NC	p	70	49/5.72	-	+	-
39	31/6.62	NC	NC	p	71	45/5.68	+	+	-
40	31/6.63	NC	NC	p	72	43/5.61	-	+	-
56	73/5.77	NC	NC	+	73	43/5.63	-	-	+
57	68/5.76	NC	NC	+	75	49/6.7	-	+	+
8	66/5.28	+	-	-	77	45/6.83	-	+	+
10	62/5.77	+	+	+	78	39/4.83	+	+	+
11	60/4.56	+	+	+	79	37/5.57	+	+	+
17	63/6.67	-	-	-	80	33/5.77	-	-	-
18	52/4.50	+	+	NC	81	31/5.50	+	+	-
31	40/5.13	-	+	+	83	30/5.82	-	+	-
35	32/4.74	-	-	-	84	28/5.86	-	-	-
44	29/5.17	+	+	-	85	30/7.28	-	+	+
46	27/5.18	+	+	-	86	30/5.00	-	-	+
49	23/4.71	+	NC	-	87	27/5.61	-	-	-
					88	53/5.68	+	+	-

The spot No refers to the respective gel. Obs., observed molecular weights (kDa) and isoelectric points, which were estimated from electrophoretic mobilities. +，upregulated expression; -，downregulated expression; NC, no change; N, detected only after treatment; ND, not determined; V, vertical clinorotation; H, horizontal clinorotation; G, hypergravity treatment.

Figure S1

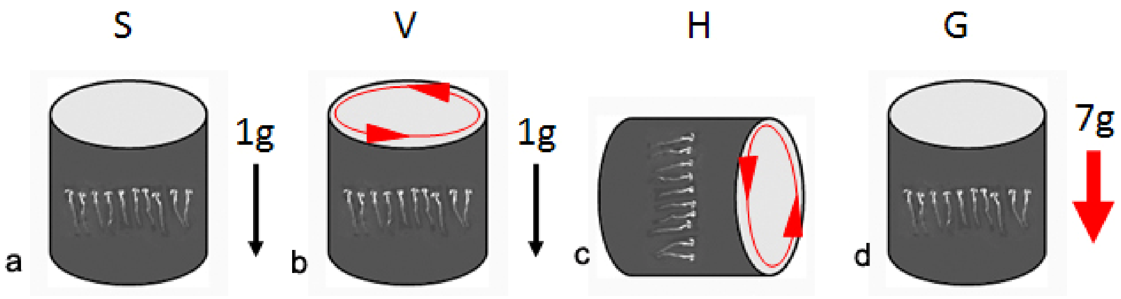

Figure S1. Schematic diagram of the experimental design for the proteomic analysis of the influence of the altered gravitational force on Arabidopsis seedlings. Seedlings were grown under 1g stationary condition (a), on a vertical (b) or horizontal (c) clinostat at 5 rpm for 12 h, or on a low-speed centrifuge to impose 7g hypergravity for 30 min (d). All the treatments were performed in the dark. S, 1g stationary control; V, vertical clinostat control; H, horizontal clinostat rotation; G, 7g hypergravity. Arrows indicate direction of gravity.

Figure S2

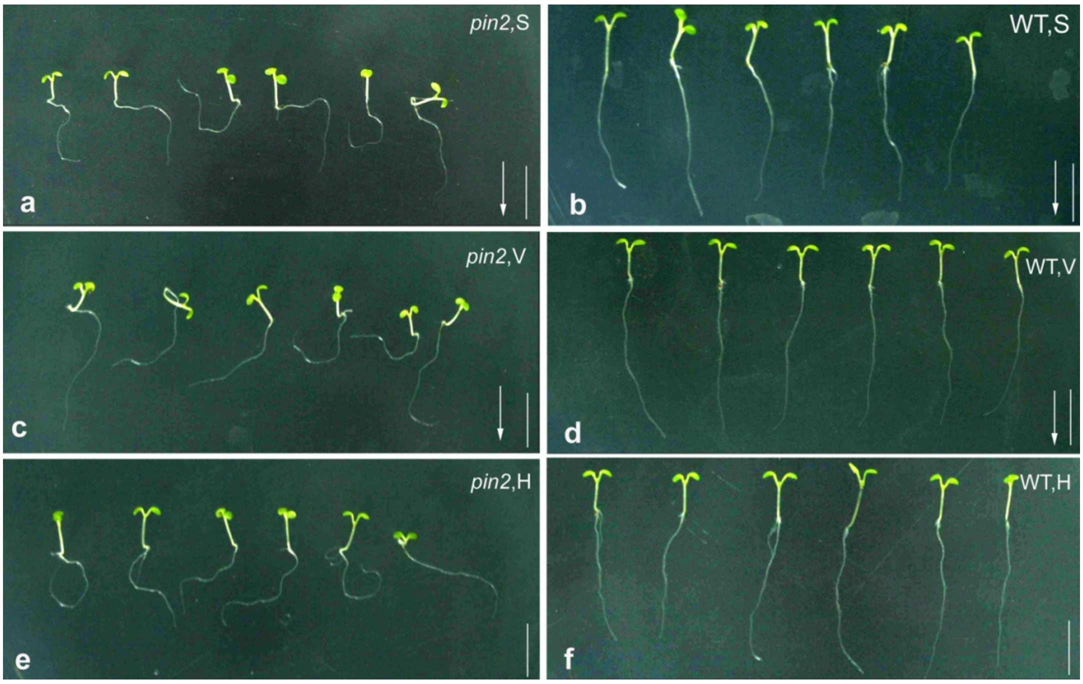

Figure S2. Six-day old Arabidopsis *pin2* mutant and wild-type (WT) seedlings grown under the 1g stationary condition (a and b) and clinorotation on a vertically (V) or horizontally (H) clinostat rotational conditions at 5 rpm for 12h (c, d, e and f). *pin2* roots are defective in gravitropic response in comparison with the normal gravitropic response of WT roots.

Figure S3

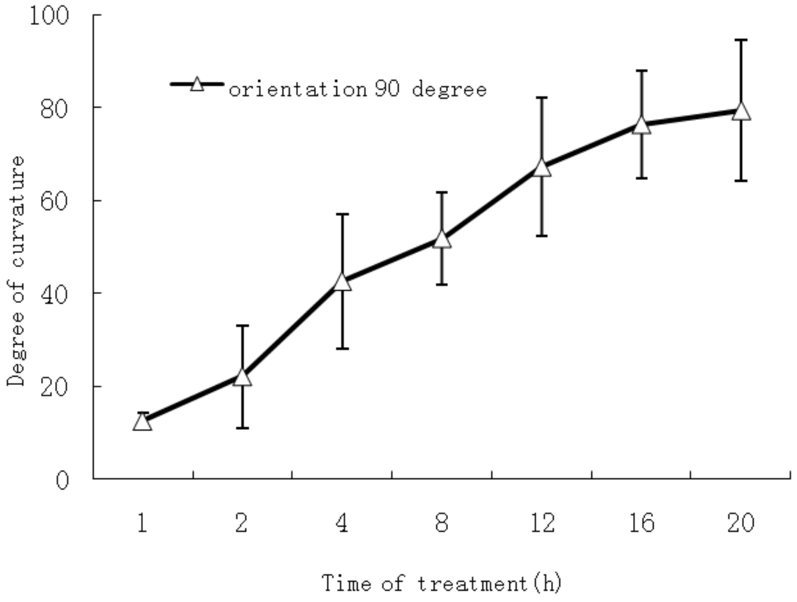

Figure S3 Statistical analysis of curvature responses of roots of six-day old Arabidopsis wild-type seedlings after rotated through 90 degree (from the vertical to the horizontal position). Data are mean±SD, n >50 roots.

Figure S4

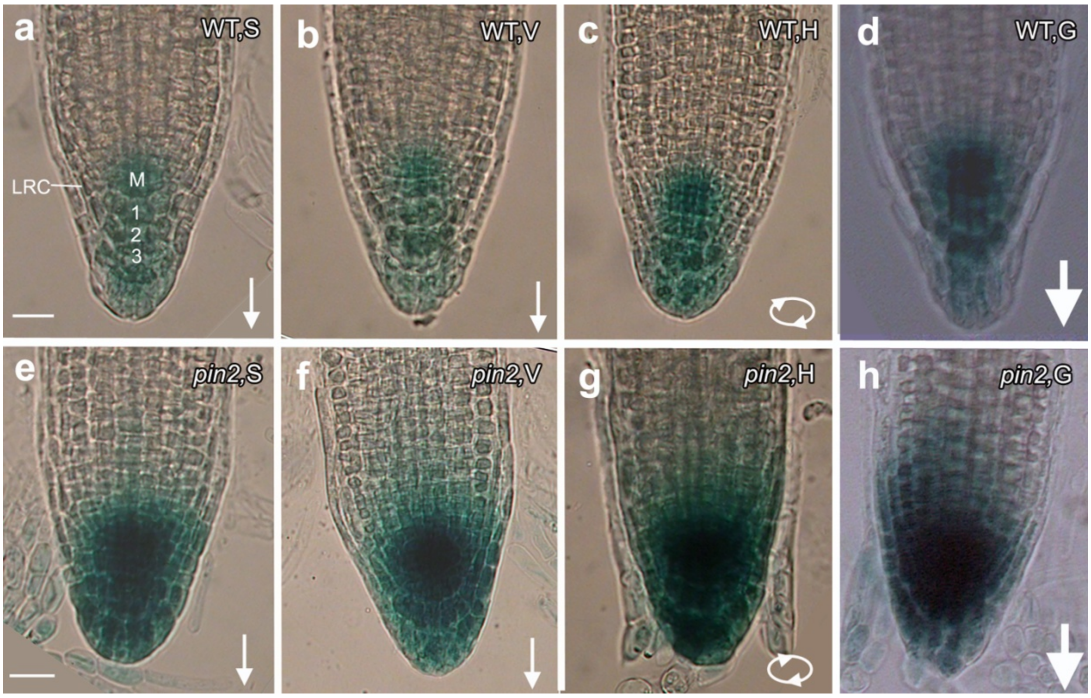

Figure S4. Expression of DR5 reporter apparently increase in *pin2* mutant root cap cells (e-f) in comparison with control of WT (a-d) under the 1g stationary condition (S), on the vertical (V) or horizontal (H) clinorotational condition at 5 rpm for 12 h, or on hypergravity (G) at 7g for 30 min . Bars=20 μm.
